# Successful Treatment of Rhabdomyolysis-Associated Acute Kidney Injury with Haemoadsorption and Continuous Renal Replacement Therapy

**DOI:** 10.1155/2021/2148024

**Published:** 2021-10-04

**Authors:** Wun Fung Hui, Kam Lun Hon, Kin Shing Lun, Karen Ka Yan Leung, Wing Lum Cheung, Alexander K. C. Leung

**Affiliations:** ^1^Department of Paediatrics and Adolescent Medicine, The Hong Kong Children's Hospital, Kowloon Bay, Hong Kong; ^2^Department of Pediatrics, The University of Calgary and The Alberta Children's Hospital, Calgary, AB T2M 0H5, Canada

## Abstract

We report two children with rhabdomyolysis-associated acute kidney injury who were successfully treated with a haemoadsorption column CytoSorb® in addition to continuous renal replacement therapy (CRRT). A 14-year-old girl with multiorgan failure requiring extracorporeal membrane oxygenation developed rhabdomyolysis due to reperfusion injury. Her creatine kinase (CK) and lactate levels continued to escalate despite high-dose CRRT. A haemoadsorption column was therefore added post-CRRT filter, which brought down the CK level from 264,500 IU/L to 97,436 IU/L after 8 hours of therapy. Another 4-year-old boy with epilepsy and cerebral palsy who was admitted for gastroenteritis with dehydration developed acute kidney injury and rhabdomyolysis with a peak CK level of 946,060 IU/L. He was initially treated with CRRT for 40 hours, which reduced his CK level to 147,580 IU/L. Two sessions of haemoadsorption were then performed in addition to the CRRT, which further lowered his CK level to 32,306 IU/L in 48 hours. Both patients demonstrated enhanced reduction of CK levels when the haemoadsorption column was used in addition to the CRRT, and no specific complication related to the haemoadsorption therapy was reported. Our cases showed that haemoadsorption can be considered as an adjunctive therapy for children with severe rhabdomyolysis-associated acute kidney injury.

## 1. Introduction

Rhabdomyolysis is characterized by the release of skeletal muscle intracellular contents including myoglobin, electrolytes, and various enzymes such as creatine kinase (CK) after muscle damage or injury. [1, 2] The pathophysiology of rhabdomyolysis involves either direct injury to the cell membrane or cellular energy depletion [2]. Acute kidney injury (AKI) is one of the most severe complications of rhabdomyolysis, which is believed to be mediated through renal vasoconstriction and myoglobinuria [3]. Myoglobin in the urine may precipitate under low pH environment, forming intratubular casts with Tamm–Horsfall protein. Myoglobinuria may also trigger the release of reactive oxygen species that exert direct toxicity via lipid peroxidation [3].

The reported incidence of AKI among children with rhabdomyolysis ranged from 5% to 36%, and around 11–36% of those with AKI required renal replacement therapy [1, 4–6]. In clinical practice, the decision of initiating renal replacement therapy is usually based on the fluid status and the severity of the metabolic complications of AKI instead of the serum concentration of CK or degree of myoglobinuria [3]. There are reports investigating various dialysis approaches for myoglobin removal. A high-dose continuous venovenous haemofiltration with replacement rate up to 3 L/hour has been advocated as one of these methods [7]. Also, the combined use of super high-flux membrane and high-dose haemofiltration has also been reported [8]. Besides haemofiltration, the role of extracorporeal blood purification technique has also been explored recently [9]. There is a paucity of information in the paediatric literature on the combined use of haemoadsorption and CRRT in the treatment of rhabdomyolysis-associated AKI. Herein, we describe our experience of using an adsorption column CytoSorb® in addition to CRRT for the treatment of rhabdomyolysis-associated AKI in two children.

## 2. Case Reports

### 2.1. Case 1

**Figure 1:**
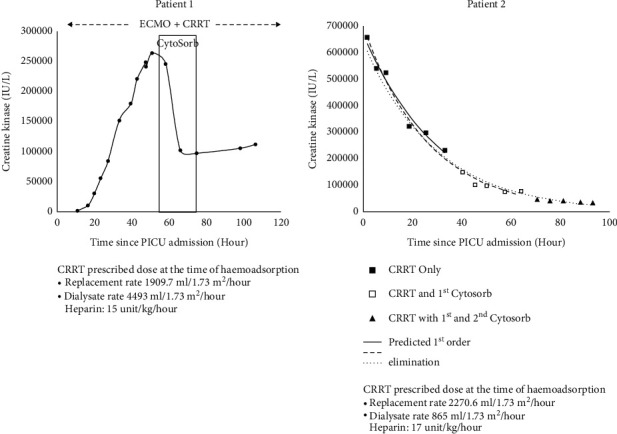
Trend of creatine kinase levels during CRRT and haemoadsorption treatment. For patient 2, the serum creatine kinase level after admission was plotted against time. A trend line using log scale was created for three different sets of data for first-order elimination prediction (black square represents data from CRRT only, white square represents data from CRRT plus 1st column of CytoSorb, and black triangle represents data from CRRT plus 1st and 2nd columns of CytoSorb). A slightly faster rate of decline could be observed from data using both CRRT and CytoSorb.

A 14-year-old girl was transferred to a paediatric intensive care unit (PICU) due to refractory shock. Her admission blood pressure was 69/34 mmHg and she rapidly developed multiorgan failure requiring high-dose inotropes, mechanical ventilation, and CRRT to support her organ function. Five hours after admission, she was started on venoarterial extracorporeal membrane oxygenation (VA-ECMO) with a flow of 5 L/min cannulated via the femoral artery and vein. Unfortunately, she developed left limb ischemia requiring revision of the reperfusion catheter, and subsequently, there was reperfusion injury causing a marked elevation of the serum CK level from 1,476 IU/L to a peak serum level of 264,500 IU/L. There was also refractory lactic acidosis with a lactate level of 15 mmol/L despite escalation of the CRRT dose (prescribed dose up to replacement rate 1,910 ml/1.73 m^2^/hour and a dialysate rate 4,493 ml/1.73 m^2^/hour). Therefore, an adsorption column CytoSorb® was added post-CRRT filter for myoglobin clearance. Heparin infusion at 15 units/kg/hour was used. Unfortunately, the circuit clotted eight hours afterwards. Despite the short treatment duration, there was dramatic reduction in her serum CK level to 97,436 IU/L after adding the haemoadsorption column (Figure 1). Except for the high return pressure on the CRRT circuit, there was no specific complication related to the haemoperfusion. At the same time, her blood smear and immunophenotyping results also came back and confirmed the diagnosis of anaplastic large cell lymphoma, and hence, dexamethasone, cyclophosphamide, and vinblastine were started. Her serum CK rebounded later owing to the ongoing reperfusion injury, but second haemoadsorption column was not employed to avoid removal of her chemotherapy. Despite all active and supportive treatment, her condition deteriorated, and she finally succumbed 5 days after admission.

### 2.2. Case 2

A 4-year-old boy with infantile epileptic encephalopathy and cerebral palsy was admitted to a general paediatric ward for norovirus-associated gastroenteritis with dehydration. There was also increased seizure frequency of up to 10 times per day before admission (baseline frequency was around 4 times per day). Blood test revealed AKI and rhabdomyolysis with a serum creatinine level of 50 *μ*mol/L (baseline creatinine level 21 *μ*mol/L) and a serum CK level of 795,770 IU/L on admission. There was also presence of myoglobin in the urine. He was treated with hyperhydration and forced alkaline diuresis without improvement, with development of oliguria, a lowest estimated glomerular filtration rate of 31.6 ml/min/1.73 m^2^ and a peak CK level reaching 946,060 IU/L. He was then transferred to the PICU of a children's hospital for further management and renal replacement therapy. Alkaline diuresis and hyperhydration were stopped after commencing CRRT, and his serum CK level dropped from 655,000 IU/L to 147,580 IU/L after 40 hours of CRRT. With an attempt to hasten the myoglobin removal, a CytoSorb® column was added at a post-filter site of the CRRT circuit 44 hours after initiating CRRT. The CRRT dosage was kept constant during the haemoadsorption (prescribed replacement rate of 2270 ml/1.73 m^2^/hour and dialysate rate of 648 ml/1.73 m^2^/hour). The serum CK level rebounded from 73,760 IU/L to 75,530 IU/L 24 hours afterwards, and hence, a new column was exchanged and used for another 24 hours. The serum CK level after two sessions of haemoadsorption was 32,306 IU/L (Figure 1). No major complication related to the haemoadsorption was noted. His renal function and rhabdomyolysis improved gradually, and dialysis support was weaned. The child required 395 hours of CRRT in total and stayed in PICU for one month. He was eventually discharged with a blood urea level of 6.2 mmol/L and a serum creatinine level of 40 *μ*mol/L.

## 3. Discussion

The use of haemoadsorption for myoglobin removal in patients with rhabdomyolysis has attracted much attention, as conventional management strategy including volume therapy, bicarbonate infusion, and even dialysis with high-flux filter cannot sufficiently manage the condition. CytoSorb® is a haemoadsorption device with a total surface area of 40,000 m^2^ made of biocompatible porous polymer beads that can eliminate hydrophobic molecules up to the size of 55 kDa [10]. The molecular weights of myoglobin and CK are 17 kDa and 80 kDa, respectively, rendering myoglobin but not CK removable by CytoSorb®.

Several reports in adult patients documented the efficacy of haemoadsorption for myoglobin removal. Dilken et al. [9] reported a 56-year-old gentleman with rhabdomyolysis who was successfully treated by CytoSorb®. However, whether the drop of CK was solely accountable by haemoadsorption has been questioned [11]. Our paediatric experience concurs with the observation in adult patients. We understand that our cases may be susceptible to the same criticism. Firstly, we could not measure myoglobin levels from our patients directly. However, the serum CK level of patient 1 kept increasing despite high-dose CRRT before the use of CytoSorb®. Also, both our patients showed a rebound of serum CK levels either after stopping the haemoadsorption or presumed saturation of the adsorption column. Secondly, we did not apply haemoadsorption in isolation but in addition to CRRT, and only after a while of CRRT treatment. As the rate of elimination of CK and myoglobin is likely concentration-dependent according to first-order kinetics, a lower level of myoglobin and CK may render the effect of removal less obvious. Despite that, our two patients still exhibited an enhanced reduction of serum CK level, a surrogate marker of myoglobin level, after the addition of haemoadsorption column. This demonstrated the potential role of haemoadsorption for the management of severe rhabdomyolysis in childhood.

Scharf et al. recently reported a series of 43 adult patients with anuric rhabdomyolysis-associated AKI who received CRRT and CytoSorb® therapy for at least 90 minutes [12]. The study showed that CytoSorb® was effective in lowering myoglobin level by a median reduction of −29.4%, but the effect was only obvious in those without ongoing rhabdomyolysis. Despite that the study lacked a control group, and the CRRT dosage during haemoadsorption was not mentioned, haemoadsorption may be considered as an adjunctive therapy in those severe cases of rhabdomyolysis requiring dialysis.

Our experience showed that haemoadsorption can be safely applied in selected paediatric patients with severe rhabdomyolysis. Currently, we consider using haemoadsorption for children with rhabdomyolysis who show suboptimal response or no response to conventional therapy with serum creatine kinase >100000 IU/L. We evaluated the clinical response every 12–24 hours after starting haemoadsorption for saturation of capacity, which may be manifested as rebound of CK level or a plateau rate of CK decline. We consider stopping haemoadsorption when the CK level approaches 15000–20000 IU/L [13], when there is no response after use of two columns, or when the clinical condition does not permit further use of haemoadsorption. Our indication of initiation and criteria of termination are clearly arbitrary and are not evidence-based. It is hoped that future studies will provide more information on the optimal timing of application, the setup of an adsorption column in relation to CRRT and ECMO circuit, the frequency of column exchange, any special precautions in paediatric patients, and whether early removal of myoglobin can translate into reducing AKI severity or even prevention of AKI.

## 4. Conclusion

Haemoadsorption can be used for the management of children with severe rhabdomyolysis-induced acute kidney injury. More studies are required to address its optimal method of application and safety profile among paediatric patients.
